# Di (2-ethylhexyl) phthalate exposure impairs meiotic progression and DNA damage repair in fetal mouse oocytes *in vitro*

**DOI:** 10.1038/cddis.2017.350

**Published:** 2017-08-03

**Authors:** Jing-Cai Liu, Fang-Nong Lai, Ling Li, Xiao-Feng Sun, Shun-Feng Cheng, Wei Ge, Yu-Feng Wang, Lan Li, Xi-Feng Zhang, Massimo De Felici, Paul W Dyce, Wei Shen

**Affiliations:** 1 College of Animal Science and Technology, Institute of Reproductive Sciences, Qingdao Agricultural University, Qingdao 266109, China; 2 Tengzhou People’s Hospital, Tengzhou 277500, China; 3 College of Life Sciences, Qingdao Agricultural University, Qingdao 266109, China; 4 College of Biological and Pharmaceutical Engineering, Wuhan Polytechnic University, Wuhan 430023, China; 5 Department of Biomedicine and Prevention, University of Rome Tor Vergata, Rome 00133, Italy; 6 Department of Animal Sciences, College of Agriculture, Auburn University, Auburn, AL 36849, USA

## Abstract

Di (2-ethylhexyl) phthalate (DEHP), is the most common member of the class of phthalates that are used as plasticizers and have become common environmental contaminants. A number of studies have shown that DEHP exposure impacts reproductive health in both male and female mammals by acting as an estrogen analog. Here, we investigated the effects of DEHP on meiotic progression of fetal mouse oocytes by using an *in vitro* model of ovarian tissue culture. The results showed that 10 or 100 *μ*M DEHP exposure inhibited the progression of oocytes throughout meiotic prophase I, specifically from the pachytene to diplotene stages. DEHP possibly impairs the ability to repair DNA double-strand breaks induced by meiotic recombination and as a consequence activates a pachytene check point. At later stages, such defects led to an increased number of oocytes showing apoptotic markers (TUNEL staining, expression of pro-apoptotic genes), resulting in reduced oocyte survival, gap junctions, and follicle assembly in the ovarian tissues. Microarray analysis of ovarian tissues exposed to DEHP showed altered expression of several genes including some involved in apoptosis and gonad development. The expression changes of some genes clustered in cell-cell communication and signal transduction, along with plasma membrane, extracellular matrix and ion channel function classes, were dependent on the DEHP concentration. Together, these results bring new support to the notion that exposure to DEHP during gestation might exert deleterious effects on ovary development, perturbing germ cell meiosis and the expression of genes involved in a wide range of biological processes including ovary development.

Di (2-ethylhexyl) phthalate (DEHP), is a compound largely used in plasticizing polyvinyl chloride resin (PVC) products worldwide.^1^ More than 10 million tons of DEHP is estimated to be used in the production of plastics and plastic-based products every year.^2^ DEHP, due to its weak non covalent link to plastic components, can be easily released into the environment and humans and animals are exposed through oral ingestion, inhalation and skin contact, after which it enters into the blood circulation.^3^

Previous studies have indicated that DEHP exposure is associated with testicular, liver, kidney and ovary tissue disease.^3, 4, 5, 6, 7^ The modes of action of DEHP are not well understood. Mechanisms may include activation of peroxisome proliferator-activated receptors (PPARs), estrogen receptors (ERs) and to a lesser extent androgen receptors (ARs). PPARs are a group of nuclear receptor proteins that function as transcription factors for genes involved in a variety of cell activities including steroidogenesis and antioxidative actions. ERs are receptors that are activated by estrogens, in particular 17*β*-estradiol (E2). Two classes of ERs have been described: nuclear/cytoplasmic estrogen receptors ERα and ERβ, and membrane estrogen receptors (mERs). Although the expression and role of PPARs in the mammalian ovary before birth are not known, ERα is known to be expressed by both primordial germ cells (PGCs)^8^ and somatic cells^9^ within sexually undifferentiated mouse gonads. DEHP effects on early postnatal and adult ovaries appear to be a consequence of activation of PPAR and/or ER-dependent pathways. It is apparent that DEHP effects on oogenesis are strictly dependent on the period of exposure, both *in vivo* and *in vitro*. Effects are seen during several developmental waypoints including during the formation and development of PGCs, entrance and progression of oocytes into meiotic prophase I, germ cell cyst breakdown during the prenatal or early postnatal period, along with primordial follicle activation and development in the adult ovary.^10, 11^

In the present paper, we utilized an *in vitro* ovarian tissue culture system, developed in our lab, to study the effects of DEHP on prenatal or early postnatal oogenesis.^12^ While it has been reported that DEHP effects folliculogenesis through impairment of oocyte meiosis, survival, and follicle assembly,^10^ the mechanisms involved remain largely unknown. Using our *in vitro* system we investigated the mechanisms of such effects and identified the gene expression profile induced by DEHP in the fetal ovary.

## Results

### Exposure of the ovary to DEHP delays oocyte progression from pachytene to diplotene

**Figure 1:**
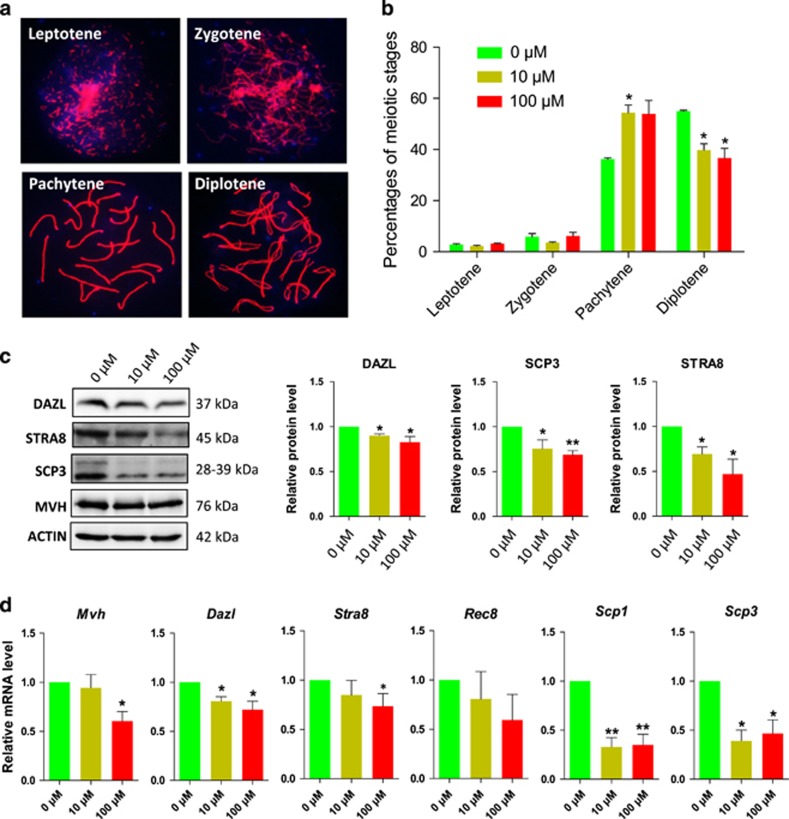
Exposure to DEHP impairs meiotic progression of oocytes from pachytene to diplotene. (**a**) Immunolabeling of the oocyte chromosomes with anti-SYCP3 antibody (red) and Hoechst 33342 (blue). (**b**) Effect of DEHP on meiotic progression of oocytes throughout prophase I stages; percentage of each group is presented as mean±SD. Control: 36.27±0.80% pachytene and 54.99±0.66% diplotene; 10 *μ*M and 100 *μ*M DEHP 57.79±4.22% and 56.62±6.62% pachytene and 39.79±4.22% and 36.62±6.62% diplotene, respectively. (**c**) Representative WB showing the effect of DEHP on the expression of germ cell (DAZL) and meiotic (STRA8 and SCP3) specific proteins. (**d**) Effect of DEHP on the levels of mRNA in the ovarian tissues of germ cell (*Mvh* and *Dazl*), meiotic (*Stra8*, *Rec8*, *Scp1* and *Scp3*). All experiments were repeated at least three times independently. (* *P*<0.05; ** *P*<0.01)

In order to explore the effects of DEHP exposure on the progression of meiotic prophase I in female germ cells, 12.5 dpc fetal mouse ovarian tissues were cultured *in vitro* and exposed to DEHP for 6 days as the first meiotic prophase I accomplished at 18.5 dpc *in vivo*, relative to day 6 *in vitro* (Supplementary Figure S1). Cytospread immunofluorescence staining of meiotic chromosomes for SCP3 revealed a clear delay in meiotic progression from the pachytene to the diplotene stage in oocytes cultured in the presence of 10 *μ*M or 100 *μ*M DEHP (Figures 1a and b). Western blot and RT-qPCR analysis showed that after 2 days of culture the expression of germ cell and meiotic genes such as *Dazl* and *Mvh* or *Stra8*, *Scp1*, *Scp3* and *Rec8*, respectively, were significantly decreased at both at the protein and/or mRNA level (Figures 1c and d).

### DEHP exposure affects the patterns of *γ*H2AX and causes DNA damage in oocytes

Since the DEHP-induced meiotic delay reported above resembles the meiotic arrest occurring in a variety of species as a consequence of the pachytene checkpoint triggered by defects in DNA double-strand break (DSB) repair,^13^ we decided to investigate the status of DNA breaks and repair in DEHP treated oocytes.

**Figure 2:**
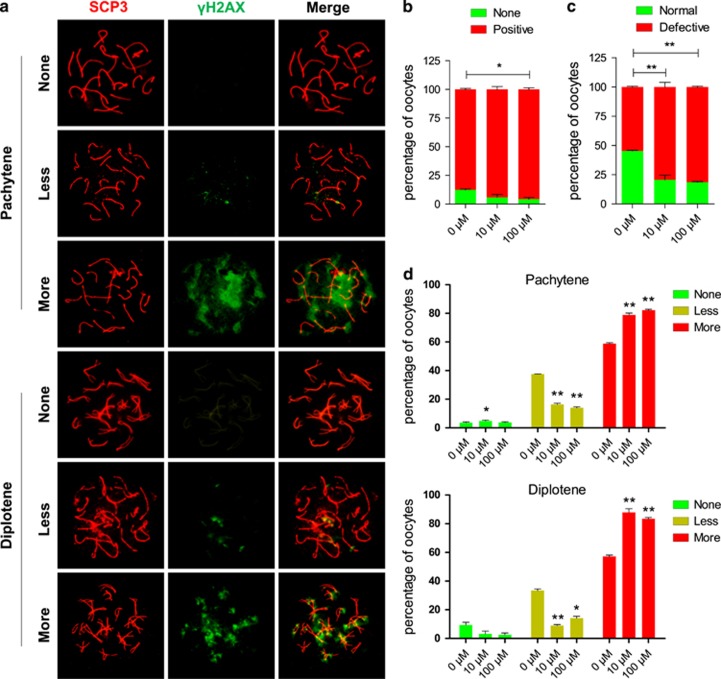
Effect of DEHP on *γ*H2AX pattern. (**a**) Immunolabeling of the oocyte chromosomes with anti-SYCP3 (red) and anti-*γ*H2AX (green) antibodies. (**b**) Percentage of positive *γ*H2AX oocytes (79.36±3.97% and 81.40±0.62%, respectively), were significantly higher than that of control group (54.70±0.61% *P*<0.01). (**c**) Percentage of normal, negative or weak *γ*H2AX staining and defective (strong *γ*H2AX staining) oocytes after six days of culture with or without DEHP. (**d**) Percentage of oocytes at the pachytene and diplotene stages showing negative, weakly and strongly positive *γ*H2AX staining after six days of culture with or without DEHP. (*P<0.05; **P<0.01)

As shown in Figure 2a, we observed that in both the control and DEHP treated oocytes the staining pattern of *γ*H2AX (a marker of DNA breaks) could be classified into three main categories: negative (none or rare barely detectable foci), weak (a few number of small foci) and strong (numerous small and large foci). As shown in Figure 2b following 6 days of culture, the percent of *γ*H2AX-positive oocytes (including weak and strong staining) was higher in DEHP treated oocytes in comparison to the control, although significantly different only in the 100 *μ*M DEHP treatment group. However, comparing negative and weak oocytes against strong *γ*H2AX stained oocytes, the difference was significant at both DEHP concentrations (Figure 2c). This difference was evident also when the analysis was restricted to pachytene and diplotene oocytes, in which the frequency of strong *γ*H2AX staining was much higher both in control and treated oocytes (Figure 2d). The presence of large *γ*H2AX foci in the DEHP exposed oocytes potentially reflects abnormal chromatin modifications during late prophase I or alternatively, a failure to resolve DSBs, and therefore a defect in DNA repair.

**Figure 3:**
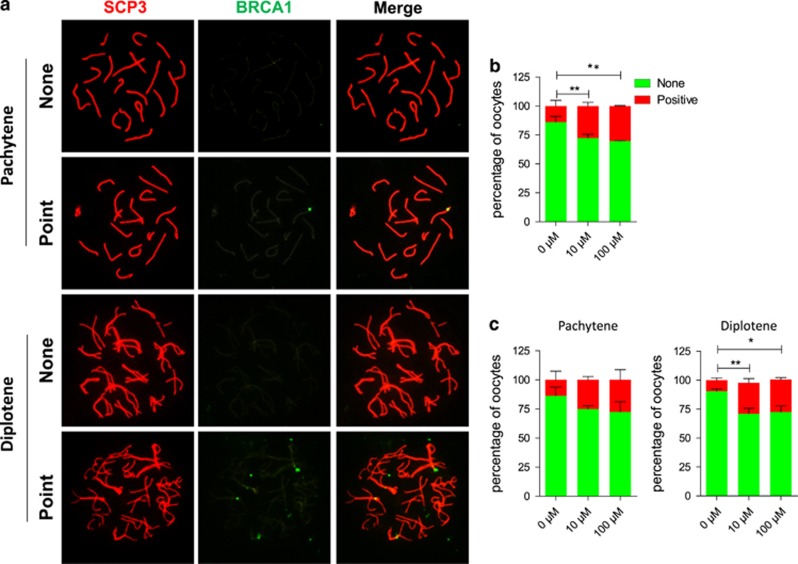
Exposure to DEHP impairs DNA repair in oocytes. (**a**) Immunolabeling of the oocyte chromosomes with anti-SYCP3 (red) and anti-BRCA1 (green) antibodies. (**b**) Percentage of BRCA1 positive oocytes after six days of culture with or without DEHP (**c**) Percentage of oocytes at the pachytene and diplotene stages showing negative or positive BRCA1 staining after 6 days of culture with or without DEHP. (*P<0.05; **P<0.01)

**Figure 4:**
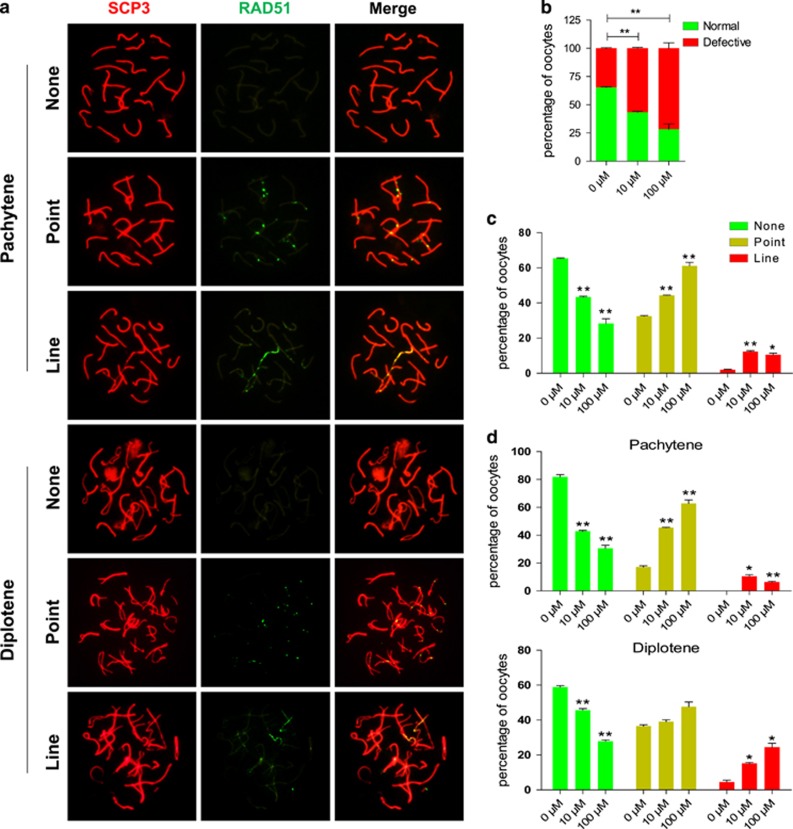
Exposure to DEHP impairs DNA repair in oocytes. (**a**) Immunolabeling of the oocyte chromosomes with anti-SYCP3 (red) and anti-RAD51 (green) antibodies. (**b**) Percentage of oocytes showing none, RAD51 staining after six days of culture with or without DEHP. (**c**) Percentage of oocytes showing none, point or line RAD51 staining after six days of culture with or without DEHP. (**d**) Percentage of oocytes at the pachytene and diplotene stages showing none, point or line RAD51 staining after six days of culture with or without DEHP (*P<0.05; **P<0.01)

Evidence of increased number of oocytes with DNA damage following DEHP exposure, were also obtained by performing chromosome staining for the proteins BRCA1, usually recruited with *γ*H2AX to sites of DNA breaks (Figure 3),^14^ and RAD51, involved in DNA repair by homologous recombination (Figure 4). Since RAD51 foci, indicative of DNA damage repair, normally disappears at the pachytene/diplotene stage oocytes at these stages still showed positive RAD51 staining both as discrete or lined dots, can be considered defective. On the other hand, the chromosome staining for the cross over protein MLH1 was not significantly different between the control and the experimental groups (Supplementary Figure S2).

Western blot results of *γ*H2AX depicted in Supplementary Figure S3A show that exposure to 10 *μ*M and 100 *μ*M DEHP for 6 days caused increased expression of *γ*H2AX in the ovarian tissue (Figure 2). Moreover, RT-qPCR analysis showed that exposure to 10 *μ*M and 100 *μ*M DEHP for 6 days caused increased expression of *Rad51*, and *Brca1* transcripts along with *Mlh1* and *Spo11* genes, the latter encoding a protein involved in the creation of DSB in the DNA at the preleptotene/leptotene stage (Supplementary Figure S3C).^15^ Finally the level of mRNA of the *Atm* gene encoding a protein that is recruited and activated by DNA breaks was not significantly altered while the expression of the *p53* gene, a gene essential for chromosome stability,^16^ was decreased both at the mRNA and protein level (Supplementary Figure S3B–C), further indicating that DEHP exposed cells accumulated DNA damage.

### DEHP promotes the expression of ERs and decreases the expression of PPAR*α*

**Figure 5:**
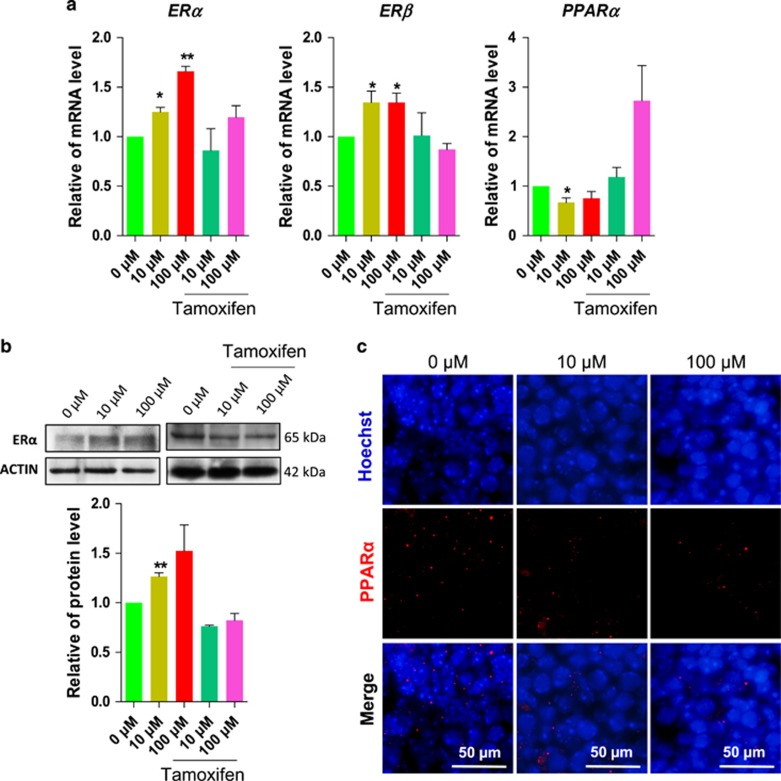
DEHP exposure affects the expression of ER and PPAR*α* in the ovary. (**a**) Representative qRT-PCR for *ERα*, *ERβ* and *PPARα* transcripts in ovarian tissues cultured for 6 days in control, DEHP and DEHP plus tamoxifen. (**b**) Representative WB of ER*α* expression. (**c**) PPAR*α*-staining of the ovarian tissues after 6 days of culture with or without DEHP. The percentage of each group is presented as mean±SD. All experiments were repeated at least three times. (*P<0.05; **P<0.01)

In order to examine the expression of ERs and PPAR*α*, two possible DEHP targets, we analyzed ovarian tissues by RT-qPCR. The analysis showed that the exposure to 10 *μ*M and 100 *μ*M DEHP for 6 days caused significantly increased mRNA expression of *ERα* and *ERβ*, and decreased the expression of *PPARα*. Such effects were partly abolished by the contemporaneous addition to the culture of 1 *μ*M tamoxifen, an ER antagonist (Figure 5a). Western blot results confirmed the increased expression of the ER*α* protein after exposure to 10 *μ*M and 100 *μ*M DEHP, and the inhibitory effect by 1 *μ*M tamoxifen (Figure 5b). Furthermore, the percentage of cells showing PPAR*α* positive staining was significantly decreased by treatment with DEHP in comparison to controls (Figure 5c).

### DEHP exposure induces apoptosis in ovarian cells

**Figure 6:**
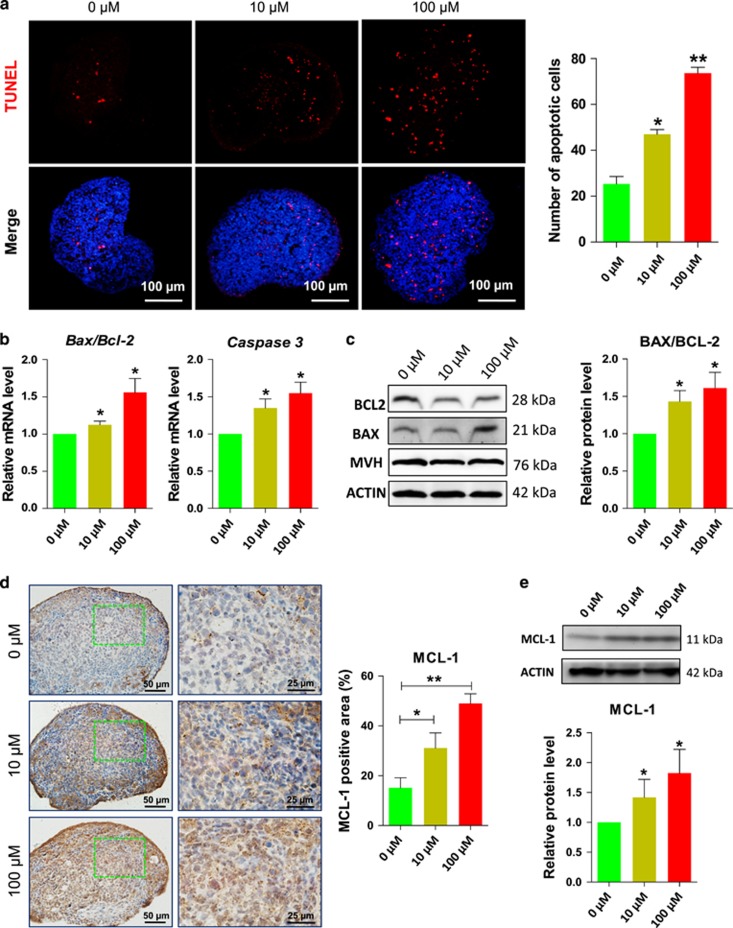
DEHP exposure promotes apoptosis in the ovary. (**a**) (Left) TUNEL-staining of the ovarian tissues after 6 days of culture with or without DEHP; (Right) Quantitative analyses of the number of TUNEL-positive cells. (**b**) Representative qRT-PCR for the apoptosis related genes *Bax*, *Bcl-2* and *Casp3* in ovarian tissues cultured for 6 days with or without DEHP. (**c**) (Left) representative WB of BAX and BCL-2; (Right) Quantitative analysis of BAX/BCL2 expression. (**d**) (Left) MCL-1-staining of the ovarian tissues after 6 days of culture with or without DEHP; (Right) Percentages of MCL-1 positive area. (**e**) (Up) Representative WB of MCL-1; (Down) Quantitative analysis of MCL-1 expression. The percentage of each group is presented as mean±SD. All experiments were repeated at least three times. (*P<0.05; **P<0.01)

Since a major consequence of unrepaired DNA damage is cell apoptosis, we analyzed ovarian tissues incubated in the presence of DEHP for 6 days for apoptotic markers. The number of cells showing TUNEL-positive staining was significantly increased by DEHP treatment when compared to the control (Figure 6a; Supplementary Figure S4). Moreover, as shown in Figure 6b, the ratio of the *Bax/Bcl-2* transcripts and the level of mRNA for the *Caspase3* gene were significantly increased in the DEHP exposed ovaries. Western blot analyses of the ovarian tissue confirmed the increased ratio of *Bax/Bcl-2* at protein level (Figure 6c), and, in addition, showed an apparent paradoxically increased expression of the anti-apoptotic protein MCL-1 after DEHP treatment in comparison to control (Figures 6d and e; Supplementary Figure S3D).

TEM images displayed some apoptotic characteristics in oocytes following DEHP treatment. While control oocytes showed a regular nuclear membrane with homogeneously dispersed chromatin and normal cytoplasmic structures, oocytes in DEHP treated groups showed condensed chromatin regions (Supplementary Figure S5A). Furthermore, numerous myelin bodies and dark homogenous osmophilic lipid droplets with distinct lamellae were observed in the 100 *μ*M DEHP treated oocytes (Supplementary Figure S5B).

### DEHP exposure reduces the number of oocytes, decreases the expression of gap junction proteins and inhibits cyst breakdown

**Figure 7:**
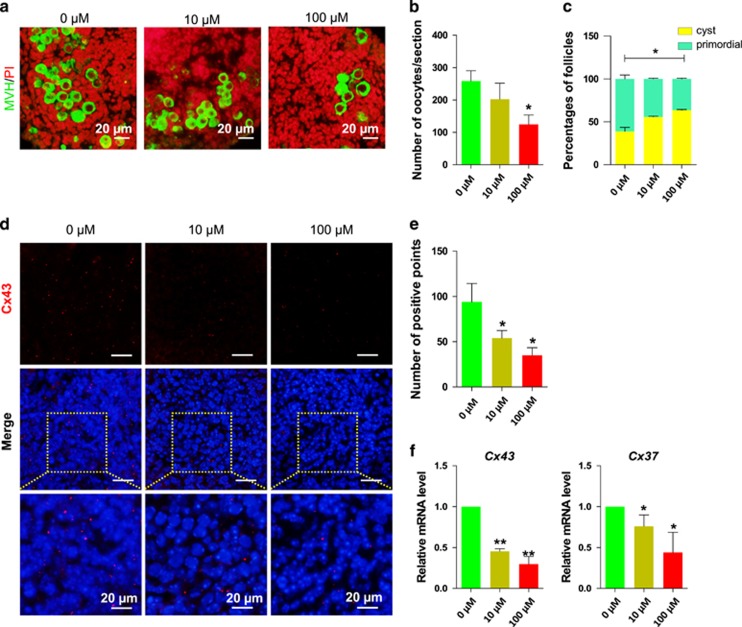
DEHP exposure causes a reduction of oocyte number and of cyst breakdown (**a**) Control and DEHP exposed ovarian tissue stained for MVH (green specific for oocytes) after 10 days DEHP exposure, nuclei red; bar is 20 *μ*M. (**b**) Number of oocytes in control and DEHP exposed groups. (**c**) Percentage of oocytes in cysts and primordial follicles in control and DEHP exposed groups. DEHP impairs the expression of connexins, (**d**) Control and DEHP exposed ovarian tissues stained for Cx43 (red), nuclei blue; bar is 20 *μ*M. (**e**) Number of Cx43 positive gap junction plaques in control and DEHP exposed ovarian tissues. (**f**) qRT-PCR for *Cx43* and *Cx37*. Relative fold changes are presented as mean±SD. All experiments were repeated at least three times. (* *P*<0.05; ** *P*<0.01)

By prolonging the culture up to 10 days we observed additional effects of DEHP exposure on the ovarian tissues. DEHP exposure caused a significant reduction in the number of oocytes (Control=258.67±31.5% 10 *μ*M DEHP=202.33±49.5% and 100 *μ*M DEHP=124.83±28.86%, Figures 7a and b).

Furthermore, a survey of the distribution of gap junctions using an antibody against Cx43, known to form channels mostly among granulosa cells, showed a decreased number of gap junctional plaques in the ovarian fragments incubated in the presence of DEHP when compared to the control unexposed fragments (Control=94.50±20; 10 *μ*M DEHP=54.25±8.26; 100 *μ*M DEHP=34.75±8.14; Figures 7d and e). RT-qPCR analyses showed a DEHP dependent reduction not only of *Cx43* expression but also of the *Cx37* gene, encoding a connexin forming gap junction channels between granulosa cells and the oocyte (Figure 7f).

Finally, numerous germ cell nests and rare follicles were detected within the ovarian tissues in the presence of DEHP (Figure 7c). Follicle counts confirmed a marked reduction in the follicle number and higher percentage of oocytes in nests in ovarian tissues cultured in the presence of DEHP (Control=61.12±8.06%, 10 *μ*M DEHP=44.20±1.55% and 100 *μ*M DEHP=36.51±1.54%).

### RNA microarray analysis of 12.5 dpc ovarian tissues exposed to DEHP

**Figure 8:**
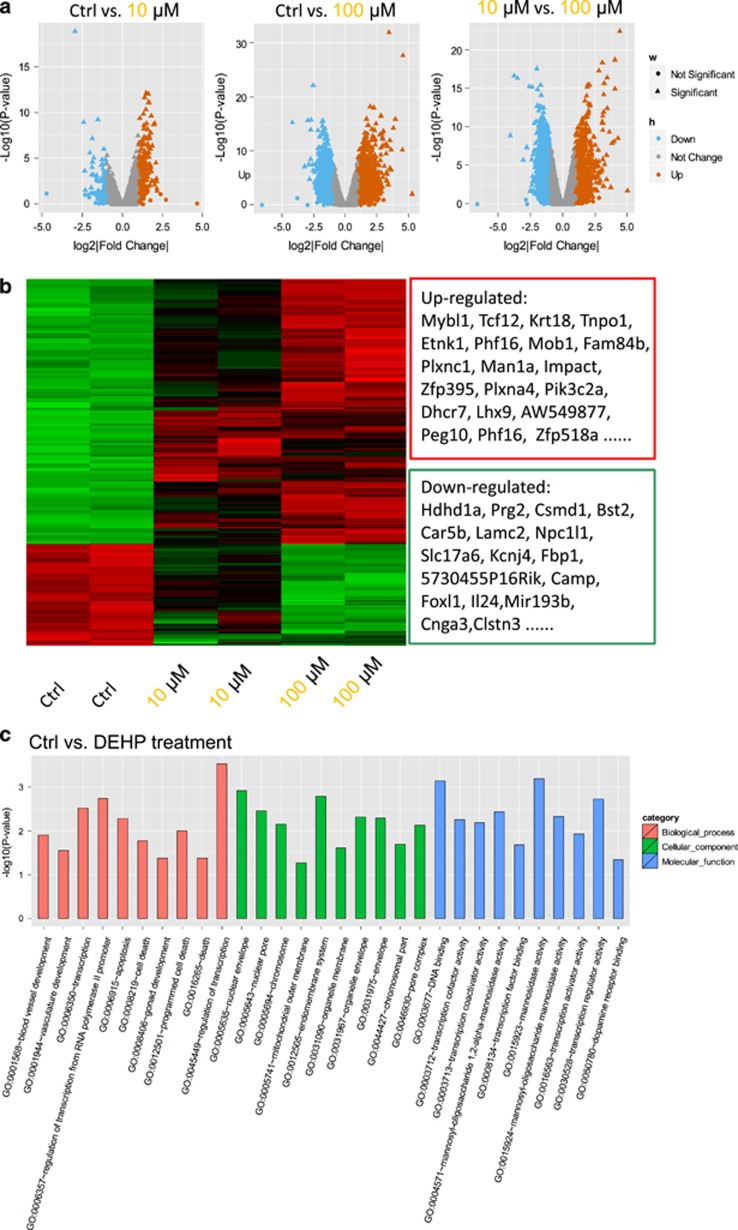
Microarray analyses of gene expression in 12.5 dpc ovary cultured for 2 days in Control (Ctr) in the presence of DEHP. (**a**) Scatter plots of differentially expressed genes between Ctr and 10 *μ*M DEHP, control and 100 *μ*M DEHP, 10 *μ*M and 100 μM DEHP groups. (**b**) Heatmap of differentially expressed genes between control and DEHP treated groups. (**c**) Gene ontology (GO) enrichment analysis of differentially expressed genes between Control (Ctr) and DEHP groups

To gain a better understanding of the biological effects of DEHP on embryonic ovaries we performed an RNA microarray analysis on 12.5 dpc ovarian tissues cultured for two days in the presence of DEHP. DEGs between the control group and DEHP exposed tissues were screened. We found a total of 206 upregulated and 54 downregulated genes following 10 *μ*M DEHP treatment, and 1529 upregulated and 1421 genes downregulated following 100 *μ*M DEHP treatment (Figures 8a and b; Supplementary Table S2). Compared to the 10 *μ*M DEHP, 1474 genes were significantly upregulated and 1505 genes were significantly downregulated in the 100 *μ*M DEHP group (Figures 8a and b; Supplementary Table S2). As a whole, 225 genes were significantly upregulated and 85 genes were significantly downregulated between control and DEHP exposed groups (Figures 8a and b; Supplementary Table S2).

Gene ontology (GO) analysis of the DEGs showed that DEHP exposure mostly perturbed genes involved in apoptosis and gonadal development (Figure 8c). As shown in Supplementary Table S2 and 3, apoptotic and gonad development genes altered after DEHP exposure included *Foxl2*, *Gm13237*, *Nkx2*-1, *Lhx9*, and *Peg10*, *Opa1*, *Krt18*, *Rock1*, *2610018G03RIK*, *Id1*, *Rhob*, *Psme3*, *Pmaip1*, *Il24*, *Phlda1*, and *Elmo1*.

Moreover, we found DEGs of DNA binding factors in both the 10 *μ*M DEHP treatment and control groups (Supplementary Figure S5A). The effects of DEHP exposure on the gene expression showed considerable dose-dependent effects. In particular, by comparing DEGs between the 10 *μ*M and 100 *μ*M DEHP groups, we found that the higher treatment mostly clustered in cell-cell communication and signal transduction, plasma membrane and extracellular matrix and ion channel functions (Supplementary Figure S5B).

## Discussion

Several studies have reported that DEHP exposure can affect the correct development and functions of the ovary. Depending on the stage of development and exposure concentration and time, both *in vivo* and *in vitro*, DEHP has been shown to alter germ cell formation and development, meiotic initiation and progression, and primordial follicle assembly.^10^ The concentrations of 10–100 *μ*M DEHP used in our study are in the range used by us and others in order to investigate the effect of this compound *in vitro* on some processes of oogenesis.^17, 18, 19^ They correspond to about 4 to 40 *μ*g/ml, a range reported in the blood of patients having a long-term exposure to DEHP-containing devices about 70 to 80 *μ*g/ml, or after neonatal exposure to DEHP following exchange transfusion with PVC catheters, between 13.2 to 84.9 *μ*g/ml.^20^

We report here that *in vitro* exposure of the mouse fetal ovaries to DEHP inhibited the progression of oocytes throughout meiosis prophase I, specifically, the transition from the pachytene to the diplotene stage. Moreover, we found that oocytes exposed to DEHP showed increased DNA damage as evaluated by *γ*H2AX and BRCA1 staining and the perdurability of RAD51 foci. As a consequence, defective oocytes could be prone to undergo apoptosis which is consistent with what we found in ovaries cultured in the presence of DEHP for six days.

Besides some morphological evidence in oocytes, we obtained indication of apoptosis in the ovarian cells following DEHP exposure from the increased *Bax/Bcl-2* ratio, *Caspase 3* and *MCL-1* expression. As a matter of fact, although MCL-1 is considered an anti-apoptotic protein, its expression has been reported to increase upon exposure to various types of apoptotic stimuli including DNA damage, likely as an attempt by cells to preserve their viability.^21, 22^

Activation of a quality check point, at the pachytene stage, which is able to eliminate oocytes carrying DNA defects has been proposed as one of the causes of oocyte depletion occurring during prenatal oogenesis in mammals.^23, 24^ Increased apoptosis explains the reduced number of oocytes scored by us after a prolonged culture period. Likewise, *in vitro* exposure for 24 h to MEHP, the active metabolite of DEHP, was shown to decrease the viability of mouse fetal oocytes, an effect attributed to an increase in oocyte oxidative stress.^25^

Since DEHP can interact with a subset of PPARs, a group of nuclear receptors that function as transcription factors for genes encoding enzymes involved in oxidative stress,^1, 26, 27^ it can be postulated that the increased DNA damage detected in cultured oocytes exposed to DEHP results from reduced activity of antioxidant enzymes. Our finding that ovarian tissues express PPARs and that such expression decreased in culture in the presence of DEHP support such a possibility. Alternatively, some of the DEHP effects reported here could be attributed to an estrogenic endocrine disruptor action. Actually, ERs are another possible DEHP targets.^10^ Two main classes of ER activated with different efficiency by E2 have been identified: ER*α* and ER*β*, which are members of the nuclear hormone family of intracellular receptors. Once activated by E2, ERs are able to translocate into the nucleus and bind to DNA to regulate the activity of different genes. It is known that ER*α* is expressed both by primordial germ cells and somatic cells of mouse sex indifferent gonads and that this expression continues to later stages while at 16.5 dpc ER*β* expression was observed in both ovaries and testes.^8, 9, 28, 29^ Here, we confirmed ER expression in the ovarian tissues regardless whether in somatic cells, oocytes or both and report an increased expression of both receptors in culture in the presence of DEHP. It is of note that in a previous study, we reported that the meiotic progression of fetal oocytes was delayed following the exposure of the pregnant mice to DEHP. This effect, resembling the present results, was associated to a reduction of the mRNA and protein expression of the meiosis-specific gene *Stra8* while the DNA methylation level of the gene was increased.^13^ The capability of DEHP to perturb methylation of meiotic genes such as *Stra8* and other genes in oocytes of neonatal mouse ovaries such as *Lhx8* and of the maternal imprinted genes *Igf2r* and *Peg3* in a transgenerational way,^17^ can be seen in the context of the contribution by ERs signaling to epigenetic changes. Several acetylases/deacetylases and methylases/demethylases can interact directly or indirectly with ER*α* and cause histone modifications.^30^ In fact, in previous studies, using the same culture system, we found that Notch signaling dependent *Stra8* methylation was necessary for correct meiotic progression.^31^ Moreover, ablation of G9a, a major mammalian H3K9 methyltransferase, showed that epigenetic gene silencing was crucial for proper meiotic prophase progression beyond the pachytene stage.^32^ Interestingly, ER*α* and ER*β* are present also in the human fetal ovary were they are present until the 20th week of gestation, when the expression of ER*α* substantially increases.^33^

It is to be mentioned here that exposure of mice from midgestation until birth to daily doses of bisphenol (BPA), a compound that like DEHP is considered an estrogenic endocrine disruptor, resulted in chromosome synaptic abnormalities and increased rates of recombination between homologous chromosomes in the mouse oocytes. Interestingly, these effects resembled those observed in mice homozygous for a targeted disruption of the gene encoding for ER*β*.^34^ Increased recombination was also observed in oocytes of rhesus monkeys prenatally exposed to BPA and in human fetal oocytes treated *in vitro* with BPA.^35, 36^ Moreover, the expression of genes involved in recombination and DNA repair and the epigenetic integrity was altered in BPA-exposed mouse and human oocytes.^7, 37, 38^

In addition to effects on meiosis, the adverse effect reported by us in the present paper on gap junction assembly and germ cell cyst breakdown can be also be attributed to an estrogenic endocrine disruptor action. In fact, estrogens are able to modulate the quantity, size and distribution of gap junctions in the ovaries and to negatively affect germ cell cyst breakdown.^17, 39, 40^

Finally, the analysis of the RNA microarray data reinforced the notion of pleiotropic dose-dependent effect of DEHP on the mammalian ovary, highlighting the possibility that this compound can affect the expression of several genes involved beside in apoptosis, in a number of process guiding prenatal gonadal development.

## Materials and methods

### Reagents

Unless otherwise indicated, all chemicals were purchased from Sigma-Aldrich (Saint Louis, MO, USA). Stock solutions of DEHP were prepared using dimethylsulfoxide (DMSO) as the solvent at concentrations of 0.127 or 1.27 M allowing an equal volume to be added to culture medium for each experimental groups. Tamoxifen, an ER antagonist, was dissolved in 50% ethanol and was added to the culture medium at the final concentrations of 1 *μ*M, and only 0.05% ethanol was present in the medium.

### Animals

The procedures involving animals followed the regulations of the Ethics Committee of Qingdao Agriculture University, Shandong, China. Mice used in this experiment were of CD1 strain purchased from Vital River Laboratory Animal Technology Co. LTD (Beijing, China). Mice were housed in 21-22 °C, 12 h dark/12 h light cycles (lights on at 7:30) with free access to food and water. Female and male mice were paired together and checked for the presence of a vaginal plug the next morning. The day when the vaginal plug was detected was considered 0.5 day post coitum (d.p.c.).

### Isolation and culture of the ovarian tissues

Isolation and culture of the ovarian tissues from 12.5 dpc embryos were performed as previously described.^12^ In particular, after isolation, the ovaries were dissected in half and each piece was cultured in 600 *μ*l of *α*-minimal essential medium (*α*-MEM; Hyclone, SH30265.01B, Beijing, China), supplemented with 10% fetal bovine serum (FBS; Gibco, 10099-141, USA), 0.23 mM sodium pyruvate (Hyclone, SH40003-12), 100 IU/ml of penicillin G, and 100 mg/ml of streptomycin sulfate, 10 mIU/ml follicle stimulating hormone (FSH; RD, 5925-FS, MN, USA). In each well, 300 *μ*l of medium was changed every two days and the culture carried out in a humidified incubator in 5% CO_2_ in air at 37 °C for the indicated time (Supplementary Figure S1).

According to United States Food and Drug Administration (FDA), tolerable intake values for DEHP are 0.6 mg/kg bodyweight/day for parenteral exposure and 0.04 mg/kg bodyweight/day for oral exposure, mouse ovaries were cultured *in vitro* and exposed to DEHP (Sigma, 36735-1G) at dose of 10 *μ*M or 100 *μ*M. DEHP was dissolved in 0.1% DMSO, and 0.1% DMSO alone as a vehicle control, respectively. After DEHP treatment, the ovaries were collected and kept for further analysis.^10^

### Staining of meiotic chromosomes

Ovarian tissues were incubated in Hypo extraction buffer (HEB; 0.6 M Tris, 0.5 M Sucrose, 0.17 M trisodium citrate dehydrate, 0.5 M EDTA, 0.5 M DTT, 0.1 M PMSF) for 1.5 h at room temperature. Tissues were then mechanically dispersed and cells transferred onto a slide fixed with 1% PFA overnight. Blocking was performed by dipping the slides in antibody dilution buffer (ADB; 0.3% BSA, 10% normal goat serum and 0.005% Triton-X-100 in TBS) for 30 min before incubation in primary antibody, anti-SCP3 at a dilution of 1:150 (Abcam, ab97672, San Francisco, CA, USA; or Novus, NB300-232, Littleton, CO, USA), anti- *γ*H2AX at a dilution of 1:150 (Abcam, ab26350), anti-RAD51 at a dilution of 1:150 (Abcam, ab133534), BRCA1 at a dilution of 1:100 (Boster, PB9015, Wuhan, China), or anti-MLH1 at a dilution of 1:150 (BD Pharmingen, 551091, Franklin Lakes, NJ, USA) for 3 h at 37 °C. The slides were further fixed with ADB overnight at 4 °C, washed three times with TBS, before incubation in secondary antibody (Beyotime, A0521 or A0516, Nantong, China; or Sigma, F0382 or F9006) at a dilution of 1:150 dilution in ADB for 3 h at 37 °C. Hoechst 33342 was used to stain nuclei and the slides were mounted with Vectashield (Vector, H-1000, Shanghai, China).^41^ Images were taken under a fluorescence microscope (BX51; Olympus, Tokyo, Japan).

### Immunofluorescence

Ovaries tissues were fixed in 4% paraformaldehyde (Solarbio, Beijing, China) for 12 h and processed for standard 5 *μ*m thick histological sections. Before staining, slides were incubated in 0.01 M sodium citrate at 96 °C for 10 min and blocked with BDT (3% BSA, 10% normal goat serum in TBS) for 45 min. and incubated with primary antibodies, rabbit anti-MVH antibody (1:150; Abcam, ab13840), rabbit anti-CX43 antibody (1:150) (Abcam, ab11370), rabbit anti-PPAR*α* (Sangon, D161086, Shanghai, China), overnight at 4 °C. After careful washing with PBS, sections were incubated with CY3-conjugated goat anti-rabbit secondary antibody at 1:150 dilution (Beyotime, A0516, Nantong, China) at 37 °C for 30 min. Vectashield (Vector) was used to mount the slides. Images were taken under a fluorescence microscope (BX51; Olympus, Tokyo, Japan).

### Immunohistochemistry

MCL-1 proteins were localization using immunohistochemistry (IHC). Put the slides in 0.01 M sodium citrate at 96 °C for 10 min, then in 3% H_2_O_2_ for 10 min and blocked with BDT (3% BSA, 10% normal goat serum in TBS) for 45 min. Rehydrated sections were incubated with MCL-1 (Sangon, D261457) primary antibodies overnight at 4 °C, then reacted with biotin-labeled secondary antibodies for 40 min at room temperature and finally stained using DAB peroxidase substrate and counterstained with hematoxylin. Image Pro Plus software 6.0 (Media Cybernetics, Rockville, MD, USA) was used to count the positive cells. In each ovary, five non-redundant sections were selected for counting and at least three ovaries per experimental group was analyzed. The data were collected from at least nine mice per treatment, and calculated the DAB peroxidase stained (positive) region, hematoxylin stained (negative) region and defined the positive region as positive stained region/(positive+negative stained regions).

### TUNEL staining

TUNEL staining was performed using the Roche “*In Situ* Cell Death Detection Kit” (Roche, 12156792910, Mannheim, Germany). Paraffin sections were heated at 60 °C for 2 h, washed in xylene and rehydrated through a series of ethanol and double distilled water. Before staining sections were treated with proteinase K for 15 min at room temperature, and carefully washed three times with PBS. 50 *μ*l of TUNEL reaction mixture (Enzyme Solution and Label Solution; 1:9 dilution) were added to the samples. For negative control, 50 *μ*l label solution (without terminal transferase) instead of TUNEL reaction mixture, was added to the slides. All slides were incubated in a humidified atmosphere for 60 min at 37 °C in the dark. Nuclei were counterstained with Hoechst 33342 (Beyotime). Images were taken under a fluorescence microscope (BX51; Olympus).

### TEM observations

For transmission electron microscopic (TEM) analyses, control and DEHP treated ovaries tissues were fixed in 2.5% glutaraldehyde in 0.1 M PBS at pH 7.2 at 4 °C for 24 h and processed following standard methods. Sectioning was performed using Leica ultra-microtome (Leica EM UC7, Wetzlar, Germany) equipped with a diamond knife. Ultrathin sections of 70 nm were collected and plated on form var-coated, carbon-stabilized copper grids, the samples were further stained with uranyl acetate and lead citrate. Samples were observed using a HITACHI HT7700 transmission electron microscope (HITACHI, Tokyo, Japan) with an accelerating voltage of 80 kV.

### Western blotting

Total proteins were extracted with RIPA lysis solution (Beyotime) for 30 min on ice with frequent vortexing. 20 *μ*l protein solution was mixed with 5 *μ*l of sodium dodecyl sulfate-polyacrylamide gel electrophoresis (SDS-PAGE) sample loading buffer and boiled for 5 min. Lysates were collected by centrifugation (12 000 rpm for 5 min). Proteins were separated by SDS-PAGE with a 4% stacking gel and a 10% separating gel for 2 h at 100 V and then transferred onto polyvinylidene fluoride membrane (18 min, 15 V) by electrophoresis. The membrane was blocked in TBST (Tris-buffered saline with Tween-20) with 10% BSA at 4 °C for 4 h and incubated with the rabbit anti-DAZL antibody (1:1000) (Abcam, ab34139), rabbit anti-STRA8 antibody (1:1000) (Abcam, ab49602), rabbit anti-SCP3 antibody (1:1000) (Novus, NB300-232), rabbit anti-MVH antibody (1:1000) (Abcam, ab13840), rabbit anti-BCL2 antibody (1:1000) (ImmunoWay, YT0470, Newark, NJ, USA), rabbit anti-BAX antibody (1:1000) (Cell Signaling Technology, 2772 Shanghai, China), rabbit anti-MCL-1 antibody (1:300), mouse anti-*γ*H2AX antibody (1:1000) (Abcam, ab26350), mouse anti- estrogen receptors (ER) *α* antibody (1:500) (Abcam, ab2746), mouse anti-*β*-actin antibody (1:1000) (Boster, BM0627) in TBST buffer containing 5% bovine serum albumin (BSA) (Solarbio, A8020) overnight at 4 °C. Then membranes were washed three times in TBST and incubated with horseradish peroxidase (HRP)-conjugated goat anti-mouse IgG (Beyotime, A0216) at 1:2000 dilution in TBST. The BeyoECL plus kit (Beyotime, P0018) was used for signal development. Beta-actin was used as the loading control. The intensity of the signal was quantified using the AlphaView SA software.

### RNA extraction and quantitative RT-PCR

Total RNA was extracted using the RNAprep pure Micro Kit (Aidlab, RN28, Beijing, China) according to the manufacturer’s instructions. Reverse transcription was performed using the TransScript One-Step gDNA Removal Kit and cDNA Synthesis Kit (TransGen Biotech, AT311, Beijing, China). All primers used in this research are listed in Supplementary Table S1. Relative quantification analysis was carried out with the LightCycler 480 II (Roche) using the LightCycler 480 SYBR Green I Master Kit (Roche, 04887352001) according to the manufacturer’s instructions. Each sample contained three technical replicates and reactions were performed with three biological replicates. The PCR conditions were as follows: 10 min at 95 °C, followed by 45 cycles of 95 °C for 10 s, 60 °C for 30 s and 72 °C for 20 s. Gene expression levels were calculated using *β-actin* for normalization. Relative transcript abundance was calculated using the 2^-ΔΔCT^ method.^41^ Data were expressed as mean±SD and calculated from at least three times independent replicates.

### RNA microarray

The protocols of total RNA microarray hybridization were consistent with our previous study.^42^ Briefly, the integrity and concentration of total RNA were measured by the Agilent 2100 Bioanalyzer (Agilent Technologies, Santa Clara, CA, USA). We used 6 *μ*g of high-quality RNA labeled with Cy5, and hybridized to a mouse oligo microarray (Phalanx Mouse Whole Genome One Array; Phalanx Biotech Group, Palo Alto, CA, USA). Each array contained 26423 DNA oligonucleotide probes of the sense strand. After hybridization, the fluorescent signals on the array were scanned using an Axon 4000 (Molecular Devices, Sunnyvale, CA, USA). Data analysis was performed based on the manufacturer’s instructions. The Differential Expression Genes (DEGs) were defined as log2 |fold change|>1 (absolute |fold change|>2) and *P*<0.05. For Gene Ontology enrichment analysis, we used the DAVID software (https://david.ncifcrf.gov/). Each array was performed using tissues from 16 ovaries for every experimental groups, and the analysis repeated at least two times.

### Statistical analysis

For each set of results, independent trials were repeated at least three times; data were represented as mean±SD. Differences among groups were statistically tested by Student’s *t*-test or one-way analysis of variance (ANOVA) multiple comparisons by using Graph-Pad Prism analysis software. Comparisons were considered significant at *P<0.05 and highly significant at **P<0.01.
