# Supplementary material for: Di (2-ethylhexyl) phthalate exposure impairs meiotic progression and DNA damage repair in fetal mouse oocytes in vitro
Source: Cell Death Dis. 2017 Aug 3;8(8):e2966–. doi: 10.1038/cddis.2017.350 (PMC5596541; doi:10.1038/cddis.2017.350)
Supplement: Supplementary Figures [file cddis2017350x1.ppt]

## Slide 1
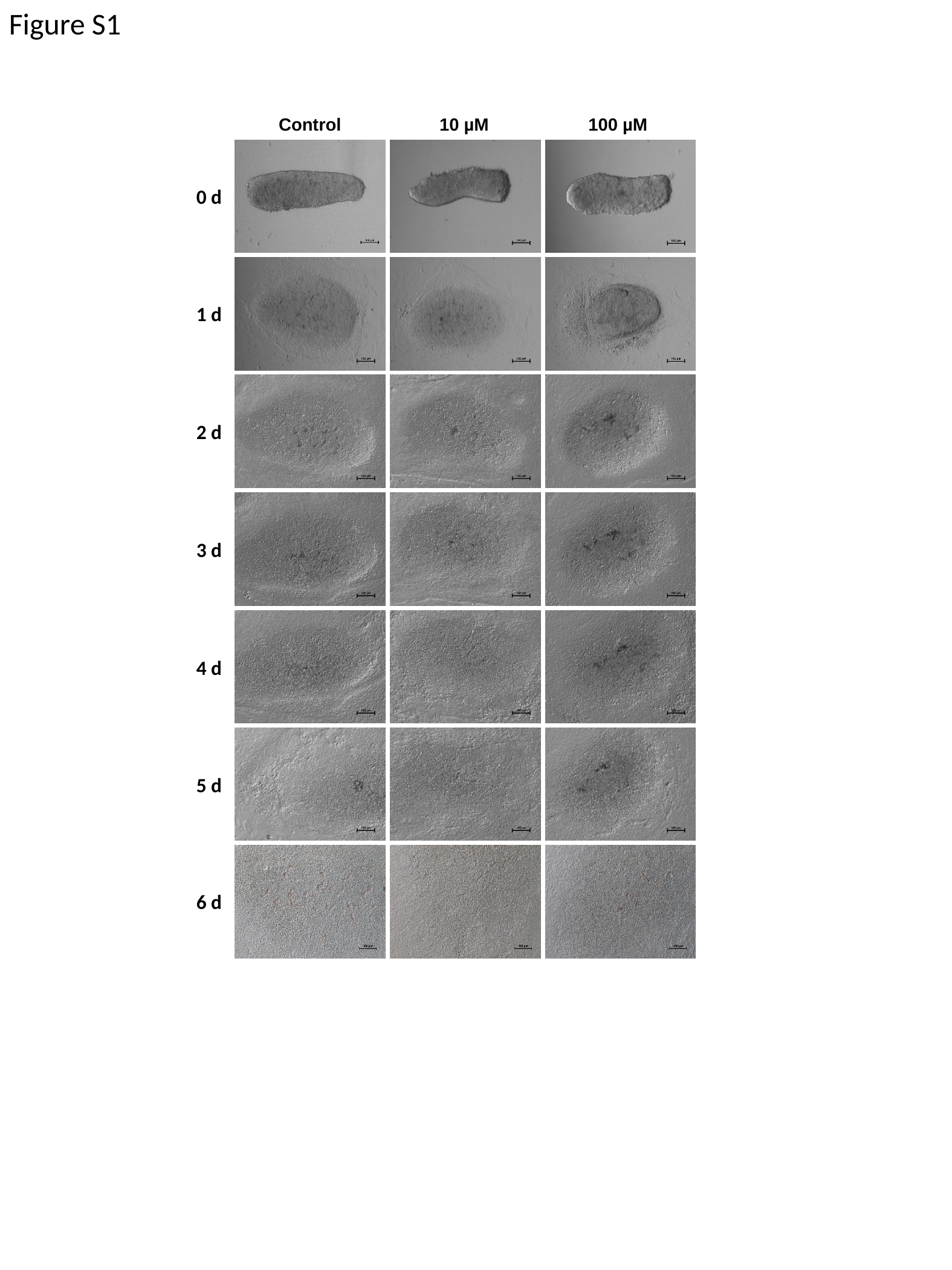

Figure S1
Control
10 µM
100 µM
0 d
1 d
2 d
3 d
4 d
5 d
6 d

## Slide 2
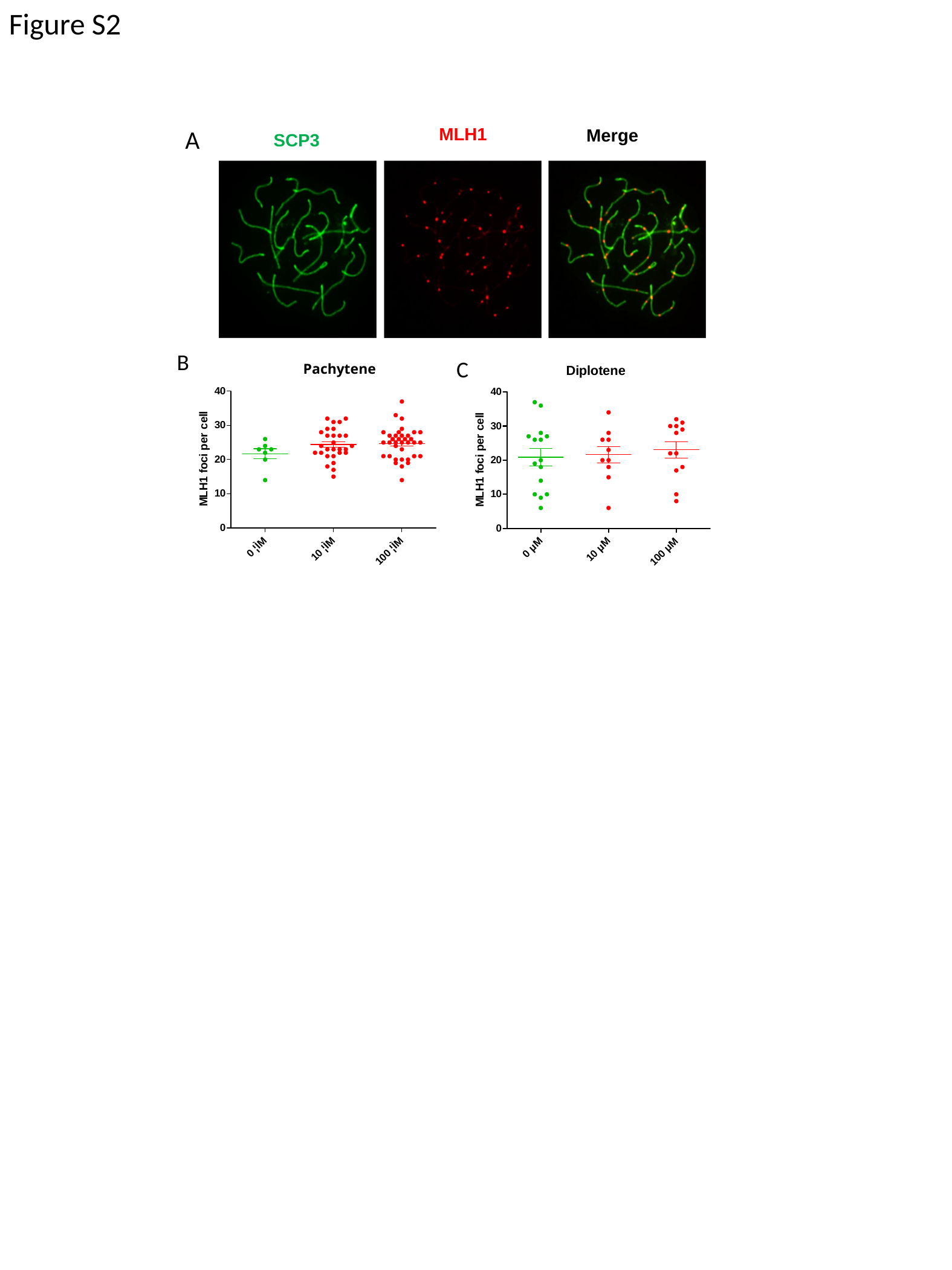

Figure S2
MLH1
Merge
SCP3
A
B
C
Pachytene

## Slide 3
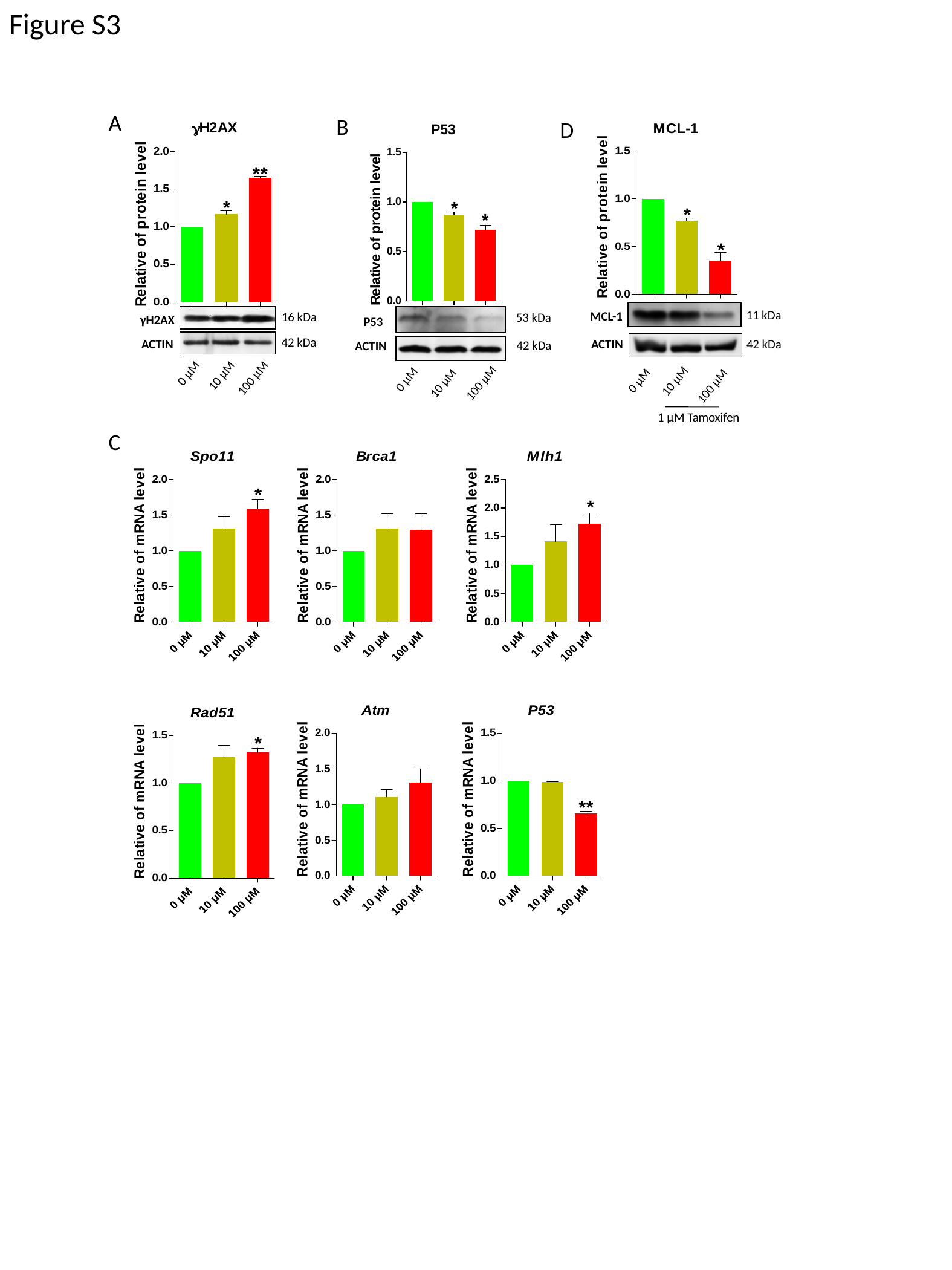

Figure S3
A
B
D
11 kDa
MCL-1
42 kDa
ACTIN
0 μM
10 μM
100 μM
1 μM Tamoxifen
16 kDa
53 kDa
γH2AX
P53
42 kDa
ACTIN
42 kDa
ACTIN
0 μM
10 μM
100 μM
0 μM
100 μM
10 μM
C

## Slide 4
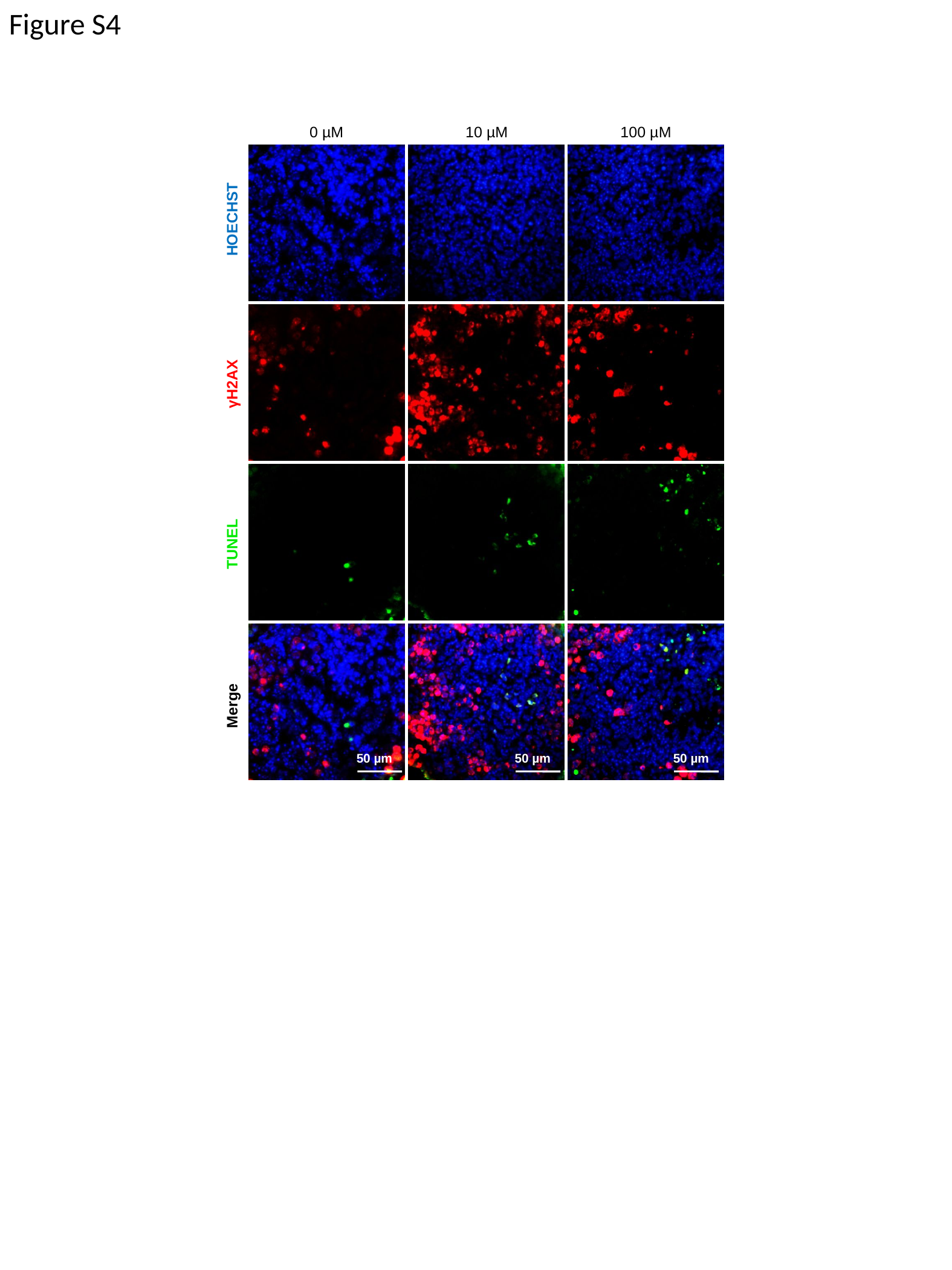

Figure S4
10 µM
100 µM
0 µM
HOECHST
γH2AX
TUNEL
Merge
50 µm
50 µm
50 µm

## Slide 5
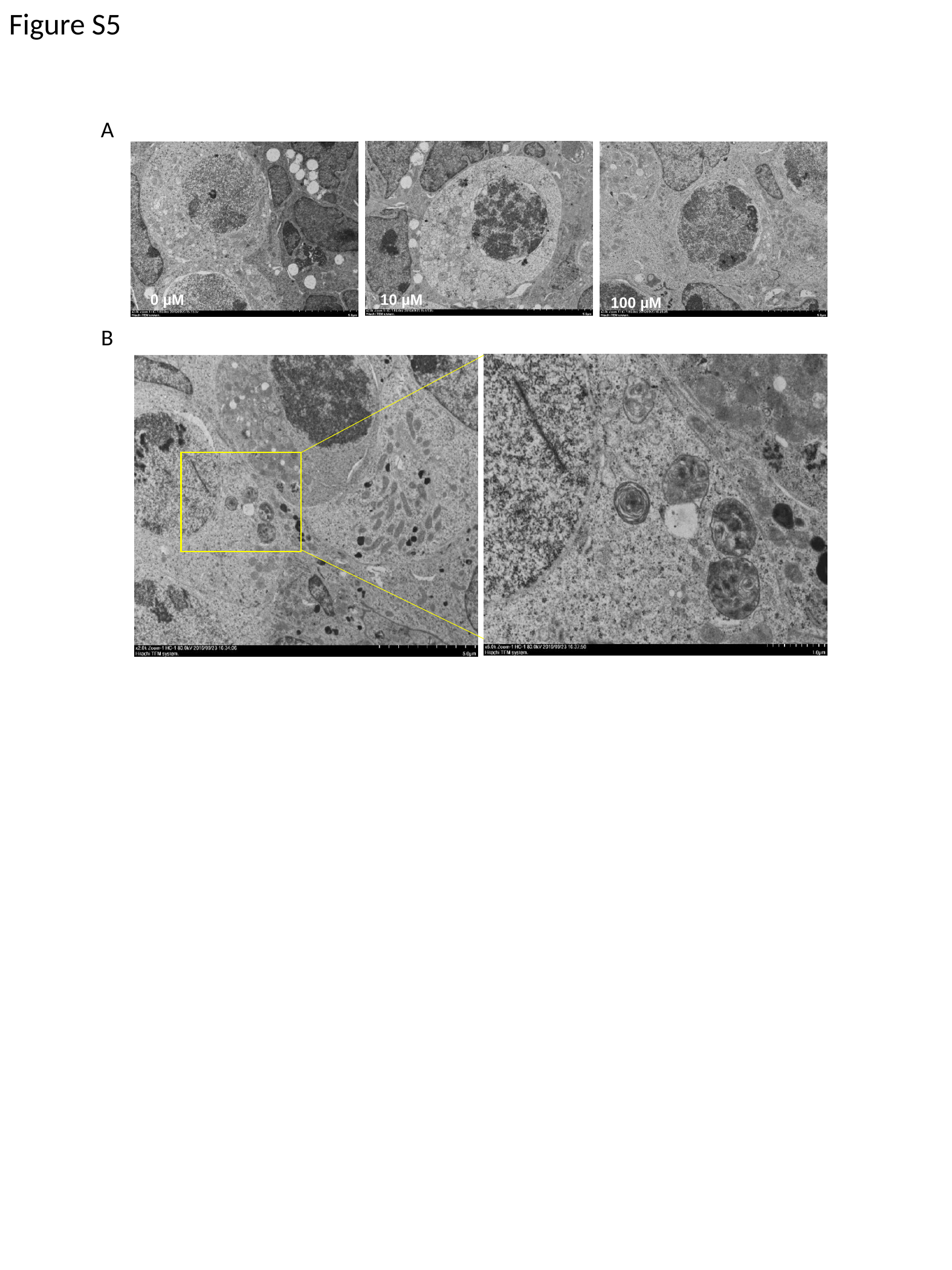

Figure S5
A
0 µM
10 µM
100 µM
B

## Slide 6
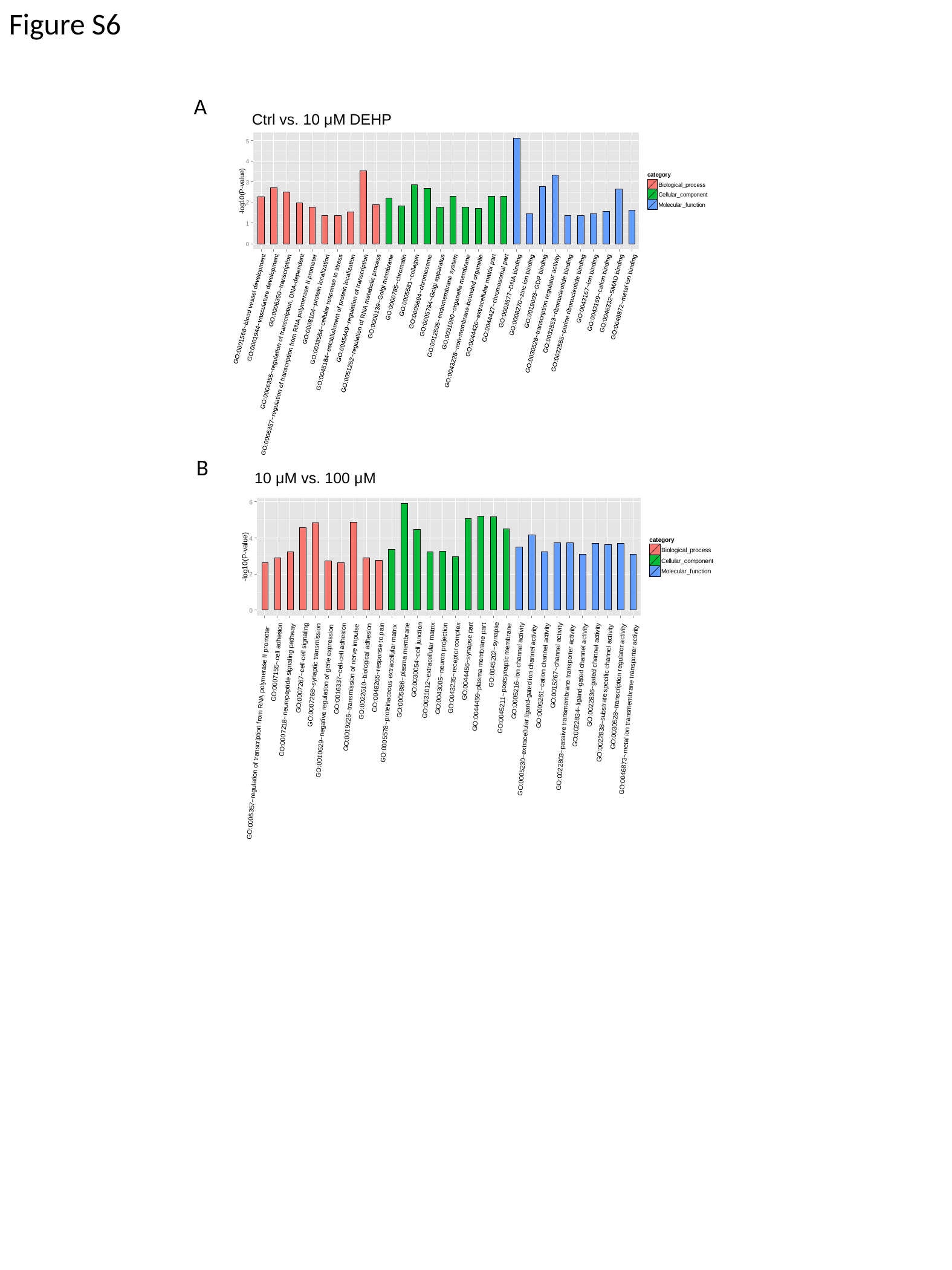

Figure S6
A
Ctrl vs. 10 μM DEHP
B
10 μM vs. 100 μM
