# Supplementary material for: Di (2-ethylhexyl) phthalate exposure impairs meiotic progression and DNA damage repair in fetal mouse oocytes in vitro
Source: Cell Death Dis. 2017 Aug 3;8(8):e2966–. doi: 10.1038/cddis.2017.350 (PMC5596541; doi:10.1038/cddis.2017.350)
Supplement: Supplementary Tables and Figure Legends [file cddis2017350x2.doc]

**Fig. S1.** Representative pictures of 12.5 dpc ovaries cultured without (Control) and with DEHP.

**Fig. S2.** Immunolocalization of MLH1 in meiotic oocytes. (A) SCP3 (red) and MLH1 (green) staining ; (B) Number of MLH1 positive oocytes at pachytene stage after DEHP exposed; (C) Number of MLH1 positive oocytes at diplotene after DEHP exposure.

**Fig. S3.** Effect of DEHP on the expression of DNA damage/repair and apoptosis related genes. The western blotting of γH2AX, p53 and MCL-1 are shown in (A, B and D); (C) Representative qRT-PCR are presented as mean ± SD. All experiments were repeated at least three times. (* P < 0.05; ** P < 0.01).

**Fig. S4.** TUNEL and γH2AX -staining of the ovarian tissues after 6 days of culture with or without DEHP. Most of the TUNEL positive signals (green) were localized on granulosa cells and the γH2AX positive signals (red) were localized on oocytes of the ovary.

**Fig. S5.** TEM evidenced apoptotic characteristics in oocytes following DEHP treatment. (A) Control oocytes showed regular nuclear membrane, homogeneously dispersed chromatin and normal cytoplasmic structures, oocytes in DEHP treated group showed a deep nuclear background. (B) Numerous myelinbodys and dark homogenous osmophilic lipid droplets with distinct lamelli were observed in 100 µM DEHP treated group.

**Fig. S6.** GO enrichment analysis of differentially expressed genes (A) between Control (Ctrl) and 10 µM DEHP group, and (B) between 10 µM DEHP group and 100 µM DEHP group.

**Table S1** Primers used for qRT-PCR

| Genes | Sequences (5'-3') | Fragment size (bp) | Accession No. |
| --- | --- | --- | --- |
| *β-actin* | F:TCGTGGGCCGCCCTAGGCAC | 243 | NM_007393.5 |
|  | R: TGGCCTTAGGGTTCAGGGGGG |  |  |
| *Bax* | F: ATGCGTCCAAGGAAGACTGAG | 162 | NM_007527 |
|  | R: CCCCAGTTGAAGTTGCCATCAG |  |  |
| *Bcl-2* | F:GCAGAGATGTCCAGTCAG | 127 | NM_009741.5 |
|  | R:CACCGAACTCAAAGAAGG |  |  |
| *Stra8* | F: CTCCTCCTCCACTCTGTTGC | 135 | NM_009292.1 |
|  | R: GCGGCAGAGACAATAGGAAG |  |  |
| *Scp3* | F: GGGGCCGGACTGTATTTACT | 169 | NM_011517.2 |
|  | R: AGGCTGATCAACCAAAGGTG |  |  |
| *Scp1* | F: GCGAAGATTGCTTTGGAGAC | 296 | NM_011516. |
|  | R: GCAGATGCCCGCAGATTAT |  |  |
| *Rec8* | F: TGATATGGAGGAGGCTGACC | 165 | NM_020002.3 |
|  | R: GCAGCCTCTAAAAGGTGTCG |  |  |
| *Dazl* | F: ATCAGCAACCACAAGTCAAGG | 192 | NM_010021.5 |
|  | R: GAGACAAATCCATAGCCCTTCG |  |  |
| *Mvh* | F: ATGATGCGGGATGGAATAACT | 476 | NM_010029.2 |
|  | R: ACTTGCCCAACAGCGACAAAC |  |  |
| *Mlh1* | F: TTGCCAACCTGCCAGATCTA | 231 | NM_026810.2 |
|  | R:ATTTGCAGCCAATCCACAGG |  |  |
| *Rad51* | F:ACCAGACCCAGCTCCTTTAC | 171 | NM_011234.4 |
|  | R:CAAGTCGAAGCAGCATCCTC |  |  |
| *Spo11* | F:TACTGCTGTGCCGACTAACA | 232 | NM_001305434.1 |
|  | R:GTAGGGATCTGCATCGACCA |  |  |
| *Brca1* | F:ATCCCGGGAAAAGCTCTTCA | 171 | NM_009764.3 |
|  | R:GGCTGCACGATCACAACTAG |  |  |
| *ER**α* | F:ACCATTGACAAGAACCGGAG | 170 | NM_007956.5 |
|  | R: CCTGAAGCACCCATTTCATT |  |  |
| *ERβ* | F: TCTTCGAAATCACCCAGACC | 138 | NM_207707.1 |
|  | R: TGTGTGTGAAGGCCATGATT |  |  |
| *PPARα* | F: ACCTTGTGTATGGCCGAGAA | 236 | NM_011144.6 |
|  | R:AAGGAGGACAGCATCGTGAA |  |  |
| *Caspase3* | F:GACTGGGATGAACCACGACCC | 205 | NM_001284409.1 |
|  | R:TCTGACTGGAAAGCCGAAAC |  |  |
| *P53* | F: ACAGTCGGATATCAGCCTCG | 159 | NM_001127233 |
|  | R: GCTTCACTTGGGCCTTCAAA |  |  |
| *Atm* | F: TCGATCTCATGAAGCCCCTC | 234 | NM_007499 |
|  | R: CAATCCGTGTGCTCTCCATG |  |  |
| *Cx43* | F: ACGGCAAGGTGAAGATGAGA | 239 | NM_010288.3 |
|  | R:GAGAGACACCAAGGACACCA |  |  |
| *Cx37* | F: ATAAAGGCACGAAGGGACCA | 167 | NM_008120.3 |
|  | R: GTCAAGTTGGCCCAGTTCTG |  |  |

**Table S2 Statistics of differentially expressed genes**

| Comparison | Up-regulated | Down-regulated |
| --- | --- | --- |
| Ctrl vs. DEHP (10 and 100 μM) | 225 | 85 |
| Ctrl vs. DEHP 10 μM | 206 | 54 |
| Ctrl vs. DEHP 100 μM | 1529 | 1421 |
| DEHP 10 μM vs. DEHP 100 μM | 1474 | 1505 |

**Table S3** Differential expression of genes related to gonadal development and apoptosis between Control and 10 µM DEHP treated groups.

| GO Term | Gene Name and Direction |
| --- | --- |
| GO:0008406 gonad development | *Amh↓, Foxl2↓, Gm13237↑, Nkx2-1↓, Lhx9↓* |
| GO:0006915 apoptosis | *Peg10↑, Opa1↑, Krt18↑, Rock1↑, 2610018G03RIK↑, Id1↓, Rhob↑, Psme3↑, Pmaip1↑, Il24↓, Phlda1↓, Elmo1↑* |
